# Supplementary material for: Ultrasound Evaluation of Onset Core Muscle Activity in Subjects with Non-Specific Lower Back Pain and Without Lower Back Pain: An Observational Case–Control Study
Source: Diagnostics (Basel). 2024 Oct 17;14(20):2310. doi: 10.3390/diagnostics14202310 (PMC11506778; doi:10.3390/diagnostics14202310)
Supplement: Supplementary file 1 [file diagnostics-14-02310-s001.zip › diagnostics-3217539-supplementary.pdf]

Supplementary Materials

Supplementary Material. Table S1. Summary of results in the reliability analysis (Muscles groups)

Table S1: Summary of results in the reliability analysis (Muscles groups)

		Examiner 1	Examiner 2	ICC (3,3)	95% CI	p	SEM	MDC95
**DPH (mm)**	Mean (SD)	3.5 (1.55)	3.63 (1.3)	0.889	0.624 to 0.971	<0.001a	0.926	2.567
**PF (mm)**	Mean (SD)	1.55 (1.87)	1.48 (1.76)	0.989	0.958 to 0.997	<0.001a	0.380	1.052
**EO (mm)**	Mean (SD)	2.2 (2.48)	2.48 (2.82)	0.982	0.888 to 0.996	<0.001a	0.424	1.175
**IO (mm)**	Mean (SD)	2.78 (0.91)	2.91 (0.89)	0.966	0.852 to 0.992	<0.001a	0.332	0.920
**TrA (mm)**	Mean (SD)	3 (2.31)	3.18 (2.59)	0.988	0.949 to 0.997	<0.001a	0.537	1.488
**LM (mm)**	Mean (SD)	13.05 (5.5)	12.85 (5.9)	0.983	0.935 to 0.996	<0.001a	1.481	4.106

a F test. DPH: Diaphragm, PF: Pelvic Floor, EO: External Oblique, IO: Internal Oblique, TrA: Transversus Abdominis, LM: Lumbar Multifidus, ICC: Intraclass Correlation Coefficient, CI: Confidence Interval, SEM: Standard Error Measurement, MDC: Minimal Detectable Changes.

Supplementary Material. Figures S1-S6. Bland-Altman plots

Figure S1. Bland-Altman plot of Diaphragm (DPH)

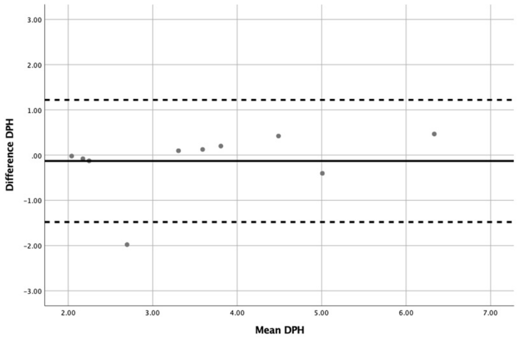

Figure S2. Bland-Altman plot of Pelvic Floor (PF)

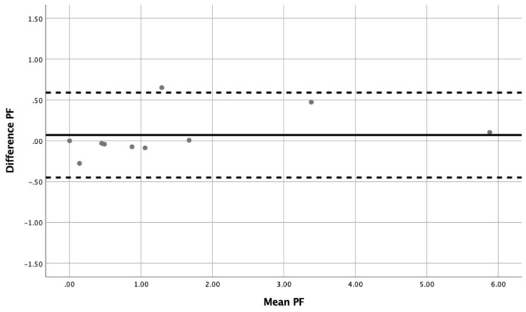

Figure S3. Bland-Altman plot of External Oblique (EO)

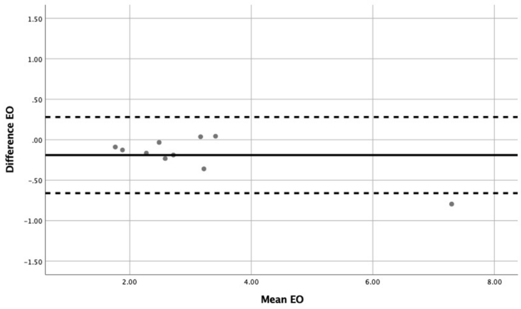

Figure S4. Bland-Altman plot of Internal Oblique (IO)

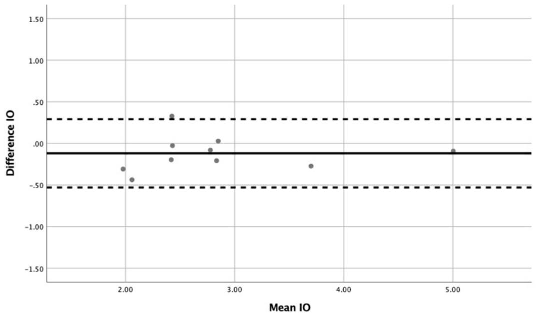

Figure S5. Bland-Altman plot of Transversus Abdominis(TRA)

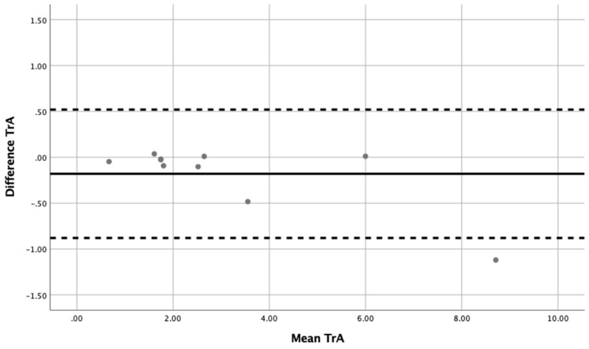

Figure S6. Bland-Altman plot of Lumbar Multifidus (LM)

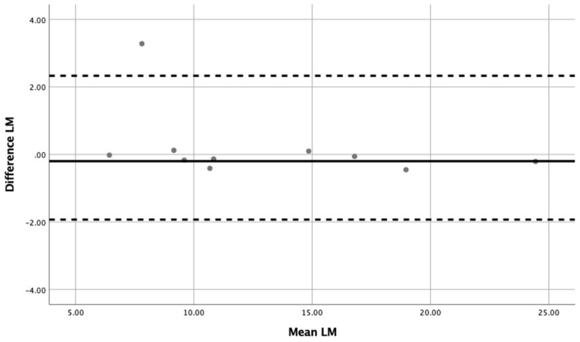

Supplementary Material. Table S2. Activation patterns identified in cases and controls in four maneuvers (ADIM, CAL, VPFC, Valsalva) in standing and sitting in order of appearances (0-40).

N.º Pattern Activation	First	Second	Third	Forth
**0**	PF	LM	LAW	DPH
**1**	PF	DPH	LAW	LM
**2**	DPH	PF	LAW	LM
**3**	LM	PF	LAW	DPH
**4**	LM	DPH	LAW	PF
**5**	DPH	LM	LAW	PF
**6**	PF	LAW	DPH	LM
**7**	PF	LAW	LM	DPH
**8**	LM	LAW	PF	DPH
**9**	LM	LAW	DPH	PF
**10**	DPH	LAW	LM	PF
**11**	DPH	LAW	PF	LM
**12**	LAW	DPH	PF	LM
**13**	LAW	PF	DPH	LM
**14**	LAW	LM	DPH	PF
**15**	LAW	PF	LM	DPH
**16**	LAW	LM	PF	DPH
**17**	LAW	DPH	LM	PF
**18**	DPH	PF	LM	LAW
**19**	LM	PF	DPH	LAW
**20**	PF	DPH	LM	LAW
**21**	PF	LM	DPH	LAW
**22**	LM	DPH	PF	LAW
**23**	DPH	LM	PF	LAW
**24**	LM	LAW	DPH	-
**25**	PF	LAW	DPH	-
**26**	LM	LAW	PF	-
**27**	DPH	LAW	LM	-
**28**	DPH	LAW	PF	-
**29**	PF	LAW	LM	-
**30**	LAW	PF	LM	-
**31**	LAW	PF	DPH	-
**32**	LAW	LM	DPH	-

**33**	LAW	LM	PF	-
**34**	LAW	DPH	LM	-
**35**	LAW	DPH	PF	-
**36**	LM	DPH	LAW	-
**37**	DPH	LM	LAW	-
**38**	DPH	PF	LAW	-
**39**	PF	LM	LAW	-
**40**	PF	DPH	LAW	-

PF: Pelvic Floor, LAW: Lateral Abdominal Wall, DPH: Diaphragm, LM: Lumbar Multifidus.

Supplementary Material. Table S3 –S10. Activation patterns: Difference in means between groups in the ADIM, CAL, VPFC and Valsalva maneuvers in the case group (Chronic non-specific low back pain) and the control group (Healthy)

Table S3. Mean difference between groups in Standing - ADIM.

		Control (n=34)	Cases (n=26)	p	Effect Size
**Standing - ADIM.**	n (%)			0.241a	0.506b
**0 (PF,LM,LAW,DPH)**		2 (5.9)	3 (11.5)		
**1 (PF,DPH,LAW,LM)**		5 (14.7)	3 (11.5)		
**2 (DPH,PF,LAW,LM)**		3 (8.8)	3 (11.5)		
**3 (LM,PF,LAW,DPH)**		1 (2.9)	1 (3.8)		
**6 (PF,LAW,DPH,LM)**		3 (8.8)	4 (15.4)		
**7 (PF,LAW,LM,DPH)**		1 (2.9)	0 (0)		
**9 (LM,LAW,DPH,PF)**		2 (5.9)	0 (0)		
**10 (DPH,LAW,LM,PF)**		2 (5.9)	1 (3.8)		
**11 (DPH,LAW,PF,LM)**		0	2 (7.7)		
**12 (LAW,DPH,PF,LM)**		6 (17.6)	0 (0)		
**13 (LAW,PF,DPH,LM)**		3 (8.8)	0 (0)		
**14 (LAW,LM,DPH,PF)**		1 (2.9)	1 (3.8)		
**15 (LAW, PF,LM,DPH)**		2 (5.9)	3 (11.5)		
**16 (LAW,LM,PF,DPH)**		0	1 (3.8)		
**17 (LAW,DPH,LM,PF)**		1 (2.9)	1 (3.8)		
**19 (LM,PF,DPH,LAW)**		1 (2.9)	0 (0)		
**20 (PF,DPH,LM,LAW)**		0	3 (11.5)		
**22 (LM,DPH,PF,LAW)**		1 (2.9)	0 (0)		

a Pearson's $\chi 2$, b Contingency coefficient. ADIM: Abdominal Drawing-In Maneuver

Table S4. Mean difference between groups in Standing - CAL.

		Control (n=34)	Cases (n=26)	p	Effect Size
**Standing - CAL**	n (%)			0.241a	0.506b
**0(PF,LM,LAW,DPH)**		0 (0)	1 (3.8)		
**1(PF,DPH,LAW,LM)**		0 (0)	2 (7.7)		
**2 (DPH,PF,LAW,LM)**		2 (5.9)	0 (0)		
**3 (LM,PF,LAW,DPH)**		0 (0)	1 (3.8)		
**4 (LM,DPH,LAW,PF)**		4 (11.8)	0 (0)		
**5 (DPH,LM,LAW,PF)**		0 (0)	1 (3.8)		
**6 (PF,LAW,DPH,LM)**		1 (2.9)	5 (19.2)		
**7 (PF,LAW,LM,DPH)**		0 (0)	2 (7.7)		
**8 (LM,LAW,PF,DPH)**		2 (5.9)	0 (0)		
**9 (LM,LAW,DPH,PF)**		2 (5.9)	3 (11.5)		
**10(DPH,LAW,LM,PF)**		2 (5.9)	2 (7.7)		
**12(LAW,DPH,PF,LM)**		1 (2.9)	0 (0)		
**14(LAW,LM,DPH,PF)**		2 (5.9)	1 (3.8)		
**15(LAW,PF,LM,DPH)**		3 (8.8)	1 (3.8)		
**16(LAW,LM,PF,DPH)**		2 (5.9)	1 (3.8)		
**18(DPH,PF,LM,LAW)**		2 (5.9)	0 (0)		
**19(LM,PF,DPH,LAW)**		1 (2.9)	0 (0)		
**20(PF,DPH,LM,LAW)**		1 (2.9)	1 (3.8)		
**21(PF,LM,DPH,LAW)**		0 (0)	1 (3.8)		
**22(LM,DPH,PF,LAW)**		2 (5.9)	1 (3.8)		
**24 (LM,LAW,DPH)**		2 (5.9)	0 (0)		

**32 (LAW,LM,DPH)**	1 (2.9)	0 (0)		
**34 (LAW,DPH,LM)**	0 (0)	1 (3.8)		
**36 (LM,DPH,LAW)**	2 (5.9)	1 (3.8)		
**37 (DPH,LM,LAW)**	2 (5.9)	1 (3.8)		

a Pearson's χ2, b Contingency coefficient. CAL: Contralateral Arm Lift

Table S5. Mean difference between groups in Standing - Valsalva.

		Control(n=34)	Cases (n=26)	p	Effect Size
**Standing - Valsalva**	n (%)			0.241a	0.506b
**1 (PF,DPH,LAW,LM)**		1 (2.9)	0 (0)		
**2 (DPH,PF,LAW,LM)**		6 (17.6)	0 (0)		
**4 (LM,DPH,LAW,PF)**		2 (5.9)	0 (0)		
**5 (DPH,LM,LAW,PF)**		1 (2.9)	2 (7.7)		
**6 (PF,LAW,DPH,LM)**		4 (11.8)	5 (19.2)		
**7 (PF,LAW,LM,DPH)**		1 (2.9)	1 (3.8)		
**9 (LM,LAW,DPH,PF)**		1 (2.9)	1 (3.8)		
**10 (DPH,LAW,LM,PF)**		1 (2.9)	1 (3.8)		
**12 (LAW,DPH,PF,LM)**		0 (0)	5 (19.2)		
**13 (LAW,PF,DPH,LM)**		2 (5.9)	2 (7.7)		
**14 (LAW,LM,DPH,PF)**		0 (0)	1 (3.8)		
**15 (LAW, PF,LM,DPH)**		3 (8.8)	1 (3.8)		
**16 (LAW,LM,PF,DPH)**		4 (11.8)	0 (0)		
**17 (LAW,DPH,LM,PF)**		2 (5.9)	2 (7.7)		
**18 (DPH,PF,LM,LAW)**		1 (2.9)	1 (3.8)		
**19 (LM,PF,DPH,LAW)**		2 (5.9)	0 (0)		
**20 (PF,DPH,LM,LAW)**		1 (2.9)	0 (0)		
**22 (LM,DPH,PF,LAW)**		0 (0)	1 (3.8)		
**23 (DPH,LM,PF,LAW)**		2 (5.9)	1 (3.8)		
**31(LAW,PF,DPH)**		0 (0)	1 (3.8)		
**35 (LAW,DPH,PF)**		0 (0)	1 (3.8)		

a Pearson's χ2, b Contingency coefficient.

Table S6. Mean difference between groups in Standing - VPFC

		Control (n=34)	Cases (n=26)	p	Effect Size
**Standing - VPFC**	n (%)			0.144a	0.555b

**1 (PF,DPH,LAW,LM)**		4 (11.8)	1 (3.8)		
**2 (DPH,PF,LAW,LM)**		1 (2.9)	1 (3.8)		
**3 (LM,PF,LAW,DPH)**		1 (2.9)	0 (0)		
**5 (DPH,LM,LAW,PF)**		1 (2.9)	0 (0)		
**6 (PF,LAW,DPH,LM)**		2 (5.9)	3 (11.5)		
**7 (PF,LAW,LM,DPH)**		3 (8.8)	4 (15.4)		
**8 (LM,LAW,PF,DPH)**		1 (2.9)	0 (0)		
**10 (DPH,LAW,LM,PF)**		1 (2.9)	0 (0)		
**11 (DPH,LAW,PF,LM)**		5 (14.7)	0 (0)		
**12 (LAW,DPH,PF,LM)**		1 (2.9)	2 (7.7)		
**13 (LAW,PF,DPH,LM)**		8 (23.5)	2 (7.7)		
**14 (LAW,LM,DPH,PF)**		1 (2.9)	0 (0)		
**15 (LAW, PF,LM,DPH)**		2 (5.9)	3 (11.5)		
**16 (LAW,LM,PF,DPH)**		0 (0)	1 (3.8)		
**17 (LAW,DPH,LM,PF)**		0 (0)	2 (7.7)		
**18 (DPH,PF,LM,LAW)**		1 (2.9)	1 (3.8)		
**19 (LM,PF,DPH,LAW)**		0 (0)	1 (3.8)		
**20 (PF,DPH,LM,LAW)**		0 (0)	4 (15.4)		
**21 (PF,LM,DPH,LAW)**		0 (0)	1 (3.8)		
**22 (LM,DPH,PF,LAW)**		1 (2.9)	0 (0)		
**25 (PF,LAW,DPH)**		1 (2.9)	0 (0)		

a Pearson's χ2, b Contingency coefficient.VPFC: Voluntary Pelvic Floor Contraction.

Table S7. Mean difference between groups in Sitting – ADIM

		**Control (n=34)**	**Cases (n=26)**	**p**	**Effect Size**
**Sitting - ADIM**	n (%)			0.802a	0.431b
**0 (PF,LM,LAW,DPH)**		1 (2.9)	1 (3.8)		
**1 (PF,DPH,LAW,LM)**		3 (8.8)	1 (3.8)		
**2 (DPH,PF,LAW,LM)**		4 (11.8)	3 (11.5)		
**6 (PF,LAW,DPH,LM)**		1 (2.9)	3 (11.5)		
**7 (PF,LAW,LM,DPH)**		1 (2.9)	0 (0)		
**8 (LM,LAW,PF,DPH)**		1 (2.9)	1 (3.8)		
**11(DPH,LAW,PF,LM)**		2 (5.9)	0 (0)		
**12 (LAW,DPH,PF,LM)**		5 (14.7)	2 (7.7)		
**13 (LAW,PF,DPH,LM)**		6 (17.6)	4 (15.4)		
**14 (LAW,LM,DPH,PF)**		0 (0)	1 (3.8)		
**15 (LAW, PF,LM,DPH)**		2 (5.9)	1 (3.8)		
**16 (LAW,LM,PF,DPH)**		1 (2.9)	0 (0)		
**17 (LAW,DPH,LM,PF)**		1 (2.9)	2 (7.7)		
**18 (DPH,PF,LM,LAW)**		1 (2.9)	1 (3.8)		
**19 (LM,PF,DPH,LAW)**		0 (0)	1 (3.8)		
**20 (PF,DPH,LM,LAW)**		1 (2.9)	1 (3.8)		
**21 (PF,LM,DPH,LAW)**		2 (5.9)	2 (7.7)		
**25 (PF,LAW,DPH)**		2 (5.9)	0 (0)		
**28 (DPH, LAW, PF)**		0 (0)	1 (3.8)		
**38 (DPH,PF,LAW)**		0 (0)	1 (3.8)		

a Pearson's χ2, b Contingency coefficient. ADIM: Abdominal Drawing-In Maneuver

Table S8. Mean difference between groups in Sitting - Valsalva.

		**Control (n=34)**	**Cases (n=26)**	**p**	**Effect Size**
**Sitting - Valsalva**	n (%)			0.241a	0.506b

**0 (PF,LM,LAW,DPH)**	0 (0)	3 (11.5)
**1 (PF,DPH,LAW,LM)**	1 (2.9)	1 (3.8)
**2 (DPH,PF,LAW,LM)**	0 (0)	3 (11.5)
**3 (LM,PF,LAW,DPH)**	0 (0)	1 (3.8)
**5 (DPH,LM,LAW,PF)**	1 (2.9)	0 (0)
**6 (PF,LAW,DPH,LM)**	3 (8.8)	2 (7.7)
**7 (PF,LAW,LM,DPH)**	1 (2.9)	0 (0)
**8 (LM,LAW,PF,DPH)**	3 (8.8)	2 (7.7)
**10 (DPH,LAW,LM,PF)**	1 (2.9)	1 (3.8)
**11 (DPH,LAW,PF,LM)**	3 (8.8)	2 (7.7)
**12 (LAW,DPH,PF,LM)**	3 (8.8)	2 (7.7)
**13 (LAW,PF,DPH,LM)**	4 (11.8)	1 (3.8)
**14 (LAW,LM,DPH,PF)**	2 (5.9)	2 (7.7)
**15 (LAW, PF,LM,DPH)**	3 (8.8)	0 (0)
**16 (LAW,LM,PF,DPH)**	1 (2.9)	0 (0)
**17 (LAW,DPH,LM,PF)**	1 (2.9)	2 (7.7)
**18 (DPH,PF,LM,LAW)**	2 (5.9)	1 (3.8)
**20 (PF,DPH,LM,LAW)**	2 (5.9)	0 (0)
**22 (LM,DPH,PF,LAW)**	1 (2.9)	0 (0)
**23 (DPH,LM,PF,LAW)**	1 (2.9)	0 (0)
**27 (DPH,LAW,LM)**	1 (2.9)	0 (0)
**32 (LAW,LM,DPH)**	0 (0)	1 (3.8)
**35 (LAW,DPH,PF)**	0 (0)	1 (3.8)
**40 (PF,DPH,LAW)**	0 (0)	1 (3.8)

a Pearson's $\chi 2$, b Contingency coefficient.

Table S9. Mean difference between groups in Sitting - CAL.

		Control (n=34)	Cases (n=26)	p	Effect Size
**Sitting - CAL**	n (%)			0.321a	0.583b
**0 (PF,LM,LAW,DPH)**		4 (11.8)	1 (3.8)		
**2 (DPH,PF,LAW,LM)**		1 (2.9)	0 (0)		
**4 (LM,DPH,LAW,PF)**		1 (2.9)	0 (0)		
**5 (DPH,LM,LAW,PF)**		0 (0)	1 (3.8)		
**6 (PF,LAW,DPH,LM)**		1 (2.9)	1 (3.8)		
**7 (PF,LAW,LM,DPH)**		0 (0)	1 (3.8)		
**8 (LM,LAW,PF,DPH)**		3 (8.8)	1 (3.8)		
**9 (LM,LAW,DPH,PF)**		4 (11.8)	0 (0)		
**10 (DPH,LAW,LM,PF)**		2 (5.9)	5 (19.2)		
**12 (LAW,DPH,PF,LM)**		2 (5.9)	0 (0)		
**13 (LAW,PF,DPH,LM)**		2 (5.9)	0 (0)		
**14 (LAW,LM,DPH,PF)**		6 (17.6)	2 (7.7)		
**15 (LAW, PF,LM,DPH)**		1 (2.9)	1 (3.8)		
**16 (LAW,LM,PF,DPH)**		1 (2.9)	0 (0)		
**17 (LAW,DPH,LM,PF)**		2 (5.9)	1 (3.8)		
**18 (DPH,PF,LM,LAW)**		0 (0)	1 (3.8)		
**19 (LM,PF,DPH,LAW)**		0 (0)	1 (3.8)		
**20 (PF,DPH,LM,LAW)**		0 (0)	1 (3.8)		
**21 (PF,LM,DPH,LAW)**		0 (0)	1 (3.8)		
**24 (LM,LAW,DPH)**		1 (2.9)	1 (3.8)		
**26 (LM,LAWPF)**		0 (0)	1 (3.8)		
**27 (DPH,LAW,LM)**		0 (0)	1 (3.8)		

**30 (LAW,PF,LM)**	0 (0)	1 (3.8)
**32 (LAW,LM,DPH)**	1 (2.9)	1 (3.8)
**33 (LAW,LM,PF)**	0 (0)	1 (3.8)
**34 (LAW,DPH,LM)**	0 (0)	1 (3.8)
**36 (LM,DPH,LAW)**	0 (0)	1 (3.8)
**37 (DPH,LM,LAW)**	1 (2.9)	0 (0)
**39 (PF,LM,LAW)**	1 (2.9)	0 (0)

a Pearson's χ2, b Contingency coefficient. CAL: Contralateral Arm Lift

Table S10. Mean difference between groups in Sitting - VPFC.

		Control (n=34)	Cases(n=26)	p	Effect Size
**Sitting - VPFC**	n (%)			0.308a	0.532b
**0 (PF,LM,LAW,DPH)**		1 (2.9)	1 (3.8)		
**1 (PF,DPH,LAW,LM)**		2 (5.9)	1 (3.8)		
**2 (DPH,PF,LAW,LM)**		1 (2.9)	1 (3.8)		
**3 (LM,PF,LAW,DPH)**		1 (2.9)	0 (0)		
**6 (PF,LAW,DPH,LM)**		6 (17.6)	4 (15.4)		
**7 (PF,LAW,LM,DPH)**		3 (8.8)	4 (15.4)		
**8 (LM,LAW,PF,DPH)**		1 (2.9)	0 (0)		
**11 (DPH,LAW,PF,LM)**		0 (0)	3 (11.5)		
**12 (LAW,DPH,PF,LM)**		5 (14.7)	1 (3.8)		
**13 (LAW,PF,DPH,LM)**		1 (2.9)	4 (15.4)		
**15(LAW, PF,LM,DPH)**		3 (8.8)	0 (0)		
**16 (LAW,LM,PF,DPH)**		1 (2.9)	0 (0)		
**17 (LAW,DPH,LM,PF)**		1 (2.9)	0 (0)		
**18 (DPH,PF,LM,LAW)**		3 (8.8)	3 (11.5)		
**19 (LM,PF,DPH,LAW)**		0 (0)	1 (3.8)		
**20 (PF,DPH,LM,LAW)**		0 (0)	1 (3.8)		
**21 (PF,LM,DPH,LAW)**		1 (2.9)	0 (0)		
**23 (DPH,LM,PF,LAW)**		0 (0)	2 (7.7)		
**25 (PF,LAW,DPH)**		1 (2.9)	0 (0)		
**29 (PF,LAW,LM)**		1 (2.9)	0 (0)		
**31 (LAW,PF,DPH)**		1 (2.9)	0 (0)		
**38 (DPH,PF,LAW)**		1 (2.9)	0 (0)		

a Pearson's χ2, b Contingency coefficient.VPFC: Voluntary Pelvic Floor Contraction.

Supplementary Material. Table S11 –S18. Muscle thickness - Difference in means between groups in the ADIM, CAL, VPFC and Valsalva maneuvers in the case group (Chronic non-specific low back pain) and the control group (Healthy).

Table S11. Average difference in muscle thickness between groups **Standing – ADIM**.

		Score difference				ANCOVA	
		**Cases (n=26)**	**Controls (n=34)**	**p**	**Size Effect**	**p**	**Size Effect**
**Diaphragm Excursion (mm)**							
**Rest**	Median (IQR)	6.68 (6.37)	5.72 (5.58)	0.245a	0.150b	-	-
**Excursion**	Median (IQR)	3.45 (14.41)	-0.62 (11.19)	**0.032a**	**0.277b**	-	-
**Pelvic Floor excursion(mm)**							
**Rest**	Median (IQR)	2.24 (3.19)	2.41 (2.37)	0.743a	0.042b	-	-
**Excursion**	Median (IQR)	12 (11.26)	8.53 (10.04)	0.314a	0.130b	-	-
**External Oblique (mm)**							
**Rest**	Median (IQR)	19.06 (12.4)	19.06 (13.98)	0.676a	0.054b	-	-
**Excursion**	Median (IQR)	18.21 (13.87)	17.65 (14.57)	0.976a	0.004b	-	-
**Ratio**	Mean (SD)	0.79 (17.67)	-3.51 (21.68)	0.412c	0.216d	0.732e	0.002f
**Internal Oblique (mm)**							
**Rest**	Median (IQR)	18.76 (16.93)	18.99 (12.56)	0.581a	0.071b	-	-
**Activation**	Median (IQR)	23.18 (21.29)	21.08 (17.38)	0.438a	0.100b	-	-
**Ratio**	Mean (SD)	30.1 (23.44)	22.83 (34.31)	0.358b	0.243d	0.891e	0.001f
**Transversus Abdominis**							
**(mm)**	Median (IQR)	12.75 (6.89)	12.28 (12.39)	0.516a	0.084b	-	-
**Rest**	Median (IQR)	19.33 (11.39)	20.84 (21.24)	0.371a	0.116b	-	-
**Activation**	Median (IQR)	47.99 (56.87)	46.24 (79.01)	0.929a	0.012b	-	-
**Ratio**							
**Lumbar Mutifidus (mm)**							
**Rest**	Median (IQR)	55.47 (37.98)	61.29 (30.29)	0.988a	0.002b	-	-
**Activation**	Median (IQR)	64.44 (36.76)	62.52 (30.57)	0.732a	0.044b	-	-
**Ratio**	Median (IQR)	4.04 (6.77)	1.93 (6.65)	**0.010a**	**0.333b**	-	-

aMann–Whitney U test, b Rosenthal's r, c Unpaired t-test, d Cohen's d, e ANCOVA, f Partial Eta-squared. ADIM: Abdominal Drawing-In Maneuver.

Table S12. Mean difference between groups in muscle thickness at Standing – CAL.

		Score difference				ANCOVA	
		**Cases (n=26)**	**Controls (n=34)**	**p**	**Effect size**	**p**	**E.Size**
**Diaphragm Excursion (mm)**							
**Rest**	Mean (SD)	6.72 (3.29)	6.08 (3.7)	0.487a	0.183b	0.684c	0.003d
**Activation**	Median (IQR)	5.17 (6.32)	4.02 (3.97)	0.835e	0.027f	-	-

**Pelvic Floor excursion (mm)**							
**Rest**	Median (IQR)	2.46 (2.28)	1.55 (2.36)	0.098e	0.214f	-	-
**Activation**	Median (IQR)	5.04 (4.48)	2.83 (3.03)	**0.012e**	**0.325f**	-	-
**External Oblique (mm)**							
**Rest**	Mean (SD)	19.25 (5.01)	18.48 (8.79)	0.742a	0.086b	0.780c	0.001d
**Activation**	Median (IQR)	19.24 (11.17)	16.73 (10.67)	0.612e	0.065f	-	-
**Ratio**	Median (IQR)	0 (12.69)	2.09 (13.77)	0.964e	0.006f	-	-
**Internal Oblique (mm)**							
**Rest**	Median (IQR)	20.90 (14.33)	17.15 (15.79)	0.210e	0.162f	-	-
**Activation**	Median (IQR)	21.11 (19.48)	20.02 (12.39)	0.318e	0.129f	-	-
**Ratio**	Median (IQR)	6.03 (21.09)	12.20 (24.66)	0.456e	0.096f	-	-
**Transversus Abdominis (mm)**							
**Rest**	Median (IQR)	11.22 (6.73)	9.58 (9.82)	1.000e	0.001f	-	-
**Activation**	Median (IQR)	13.18 (8.71)	11.19 (12.47)	0.958e	0.007f	-	-
**Ratio**	Median (IQR)	14.02 (33.32)	9.01 (47.06)	0.571e	0.073f	-	-
**Lumbar Mutifidus (mm)**							
**Rest**	Median (IQR)	65.32 (41.03)	59.99 (31.99)	0.531e	0.081f	-	-
**Activation**	Median (IQR)	70.37 (42.57)	63.91 (34.14)	0.447e	0.098f	-	-
**Ratio**	Median (IQR)	8.67 (8.69)	6.55 (9.15)	0.602e	0.067f	-	-

a Unpaired t-test, b Cohen's d, c ANCOVA, d Partial Eta-squared, e Mann–Whitney U test, f Rosenthal's r. CAL: Contralateral Arm Lift.

Table S13. Mean difference between groups in muscle thickness at Standing – VALSALVA.

		Score difference				ANCOVA	
		**Cases (n=26)**	**Controls (n=34)**	**p**	**Effect size**	**p**	**Effect size**
**Diaphragm Excursion (mm)**							
**Rest**	Median (IQR)	7.28 (6.48)	5.54 (5.41)	0.408a	0.107b	-	-
**Activation**	Median (IQR)	4.46 (11.38)	4.77 (12.36)	0.946a	0.009b	-	-
**Pelvic Floor excursion(mm)**							
**Rest**	Median (IQR)	2.00 (2.60)	1.73 (2.40)	0.676a	0.054b	-	-
**Activation**	Median (IQR)	-4.50 (14.62)	-2.39 (13.97)	0.576a	0.072b	-	-
**External Oblique (mm)**							
**Rest**	Mean (SD)	19.33 (8.69)	19.09 (8.39)	0.912c	0.029d	0.261e	0.023f
**Activation**	Mean (SD)	16.84 (7.78)	16.74 (9.14)	0.965c	0.011d	0.110e	0.045f
**Ratio**	Median (IQR)	-17.17 (26.70)	-11.93 (30.52)	0.732a	0.044b	-	-
**Internal Oblique (mm)**							
**Rest**	Median (IQR)	18.55 (21.04)	18.11 (16.18)	0.531a	0.081b	-	-
**During test**	Median (IQR)	21.02 (25.11)	21.31 (20.05)	0.835a	0.027b	-	-
**Ratio**	Median (IQR)	24.89 (40.35)	30.50 (61.10)	0.296a	0.135b	-	-

**Transversus Abdominis (mm)**	Median (IQR)	9.54 (8.08)	12.02 (9.15)	0.114a	0.204b	-	-
**Rest**	Median (IQR)	15.82 (9.09)	16.97 (17.88)	0.239a	0.152b	-	-
**Activation**	Median (IQR)	47.36 (105.59)	44.96 (102.00)	0.917a	0.013b	-	-
**Ratio**							
**Lumbar Mutifidus (mm)**							
**Rest**	Median (IQR)	60.83 (33.36)	56.19 (44.08)	0.817a	0.030b	-	-
**Activation**	Median (IQR)	68.97 (41.78)	57.53 (44.98)	0.332a	0.125b	-	-
**Ratio**	Median (IQR)	8.34 (18.72)	6.21 (9.90)	0.283a	0.139b	-	-

aMann–Whitney U test, b Rosenthal's r, c Unpaired t-test, d Cohen's d, e ANCOVA, f Partial Eta-squared.

Table S14. Mean difference between groups in muscle thickness at Standing – VPFC.

		**Score difference**				**ANCOVA**	
		**Cases (n=26)**	**Controls (n=34)**	**p**	**Effect size**	**p**	**Effect size**
**Diaphragm Excursion (mm)**							
**Rest**	Median (IQR)	6.48 (5.20)	6.23 (6.35)	0.521a	0.083b	-	-
**Activation**	Median (IQR)	2.38 (6.84)	3.62 (10.24)	0.148a	0.187b	-	-
**Pelvic Floor excursion(mm)**							
**Rest**	Median (IQR)	2.05 (3.92)	1.71 (2.56)	0.182a	0.172b	-	-
**Activation**	Median (IQR)	13.75 (18.79)	10.61 (11.39)	0.116a	0.203b	-	-
**Oblicuo Externo (mm)**	Mean (SD)	20.34 (9.83)	19.59 (9.64)	0.767c	0.078d	0.839e	0.001f
**Rest**	Mean (SD)	19.17 (8.87)	17.12 (8.26)	0.359c	0.243d	0.616e	0.005f
**Activation**	Median (IQR)	-5.98 (21.10)	-4.78 (24.83)	0.469a	0.093b	-	-
**Ratio**							
**Internal Oblique (mm)**							
**Rest**	Median (IQR)	20.52 (21.34)	15.63 (14.18)	0.245a	0.150b	-	-
**Activation**	Median (IQR)	23.88 (22.27)	19.02 (15.42)	0.210a	0.162b	-	-
**Ratio**	Median (IQR)	12.80 (30.23)	13.72 (45.97)	0.777a	0.037b	-	-
**Transversus Abdominis (mm)**							
**Rest**	Median (IQR)	12.54 (9.10)	9.57 (7.44)	0.581a	0.071b	-	-
**Activation**	Median (IQR)	15.73 (9.72)	14.38 (14.87)	0.465a	0.094b	-	-
**Ratio**	Median (IQR)	31.34 (48.96)	37.23 (95.41)	0.347a	0.121b	-	-
**Lumbar Mutifidus (mm)**							
**Rest**	Median (IQR)	58.87 (29.60)	56.13 (39.23)	0.687a	0.052b	-	-
**Activation**	Median (IQR)	62.83 (31.70)	58.63 (41.06)	0.654a	0.058b	-	-
**Ratio**	Median (IQR)	3.69 (8.39)	2.90 (7.47)	0.602a	0.067b	-	-

[A]Mann–Whitney U test, b Rosenthal's r, c Unpaired t-test, d Cohen's d, e ANCOVA, f Partial Eta-squared. VPFC: Voluntary Pelvic Floor Contraction.

Table S15. Mean difference between groups in muscle thickness at Sitting – ADIM.

		**Score difference**				**ANCOVA**	

		Cases (n=26)	Controls (n=34)	p	Effect size	p	Effect size
**Diaphragm Excursion (mm)**							
**Rest**	Median (IQR)	5.67 (6.46)	7.56 (6.55)	0.870a	0.021b	-	-
**Activation**	Median (IQR)	3.82 (15.15)	2.15 (10.29)	0.521a	0.083b	-	-
**Pelvic Floor excursion(mm)**						-	-
**Rest**	Median (IQR)	3.42 (3.23)	2.18 (2.93)	0.056a	0.247b	-	-
**Activation**	Median (IQR)	13.41 (12.85)	8.22 (9.11)	0.270a	0.143b		
**Extenal Oblique (mm)**	Median (IQR)	19.13 (17.07)	14.18 (8.50)	0.239a	0.152b	-	-
**Rest**	Median (IQR)	15.31 (13.03)	14.36 (8.74)	0.607a	0.066b	-	-
**Activation**	Mean (SD)	-6.48 (22.39)	4.25 (20.13)	0.056c	0.512d	0.282e	0.021f
**Ratio**							
**Internal Oblique (mm)**	Median (IQR)	18.63 (19.78)	17.12 (13.44)	0.660a	0.057b	-	-
**Rest**	Median (IQR)	20.23 (19.37)	17.25 (22.25)	0.581a	0.071b	-	-
**Activation**	Median (IQR)	18.02 (45.76)	15.08 (58.03)	0.748a	0.041b	-	-
**Ratio**							
**Transerso Abdominal (mm)**							
**Rest**	Median (IQR)	11.86 (7.90)	11.08 (7.68)	0.715a	0.047b	-	-
**Activation**	Mean (SD)	18.09 (8.67)	20.77 (11.58)	0.329c	0.258d	0.799e	0.001f
**Ratio**	Median (IQR)	44.39 (66.76)	60.06 (64.16)	0.612a	0.065b	-	-
**Lumbar Mutifidus (mm)**							
**Rest**	Mean (SD)	61.55 (23.62)	66.87 (27.54)	0.435c	0.206d	0.751e	0.002f
**Activation**	Mean (SD)	64.05 (24.54)	69.35 (27.77)	0.445c	0.201d	0.825e	0.001f
**Ratio**	Median (IQR)	2.99 (5.06)	1.95 (7.47)	0.617a	0.065b	-	-

aMann–Whitney U test, b Rosenthal's r, c Unpaired t-test, d Cohen's d, e ANCOVA, f Partial Eta-squared. ADIM: Abdominal Drawing-In Maneuver.

Table S16. Mean difference between groups in muscle thickness at Sitting – CAL.

		Score difference				ANCOVA	
		**Cases (n=26)**	**Controls (n=34)**	**p**	**Effect size**	**p**	**Effect size**
**Diaphragm Excursion (mm)**		5.91 (3.70)	5.67 (4.47)	0.771a	0.038b	-	-
**Rest**	Median (IQR)	6.85 (9.44)	6.08 (8.34)	0.732a	0.044b	-	-
**Activation**	Median (IQR)						
**Pelvic Floor**							
**excursion(mm)**	Median (IQR)	3.69 (3.33)	2.36 (2.47)	0.073a	0.231b	-	-
**Rest**	Median (IQR)	3.93 (5.14)	2.85 (4.13)	0.293a	0.136b	-	-
**Activation**							
**Extenal Oblique**							
**(mm)**	Median (IQR)	17.84 (18.37)	15.13 (18.96)	0.823a	0.029b	-	-
**Rest**	Median (IQR)	17.28 (19.24)	15.39 (17.91)	0.800a	0.033b	-	-
**Activation**	Median (IQR)	0 (12.29)	-0.98 (18.19)	0.512a	0.085b	-	-
**Ratio**							

**Internal Oblique**							
**(mm)**	Median (IQR)	20.65 (16.54)	15.31 (13.30)	0.152a	0.185b	-	-
**Rest**	Median (IQR)	22.70 (18.55)	14.21 (13.78)	0.053a	0.249b	-	-
**Activation**	Median (IQR)	13.14 (25.93)	10.98 (23.37)	0.318a	0.129b	-	-
**Ratio**							
**Transversus Abdominis (mm)**							
**Rest**	Median (IQR)	13.02 (6.82)	9.27 (11.77)	0.321a	0.128b	-	-
**Activation**	Median (IQR)	14.11 (9.88)	12.19 (17.81)	0.905a	0.015b	-	-
**Ratio**	Median (IQR)	0 (26.27)	23.58 (50.29)	0.033a	0.275b	-	-
**Lumbar Mutifidus (mm)**							
**Rest**	Mean (SD)	65.96 (24.34)	69.69 (31.20)	0.617c	0.132d	0.513e	0.008f
**Activation**	Mean (SD)	70.91 (25.21)	74.99 (33.08)	0.603c	0.137d	0.645e	0.004f
**Ratio**	Median (IQR)	6.95 (8.29)	6.83 (5.73)	0.917a	0.013b	-	-

aMann–Whitney U test, b Rosenthal's r, c Unpaired t-test, d Cohen's d, e ANCOVA, f Partial Eta-squared. CAL: Contralateral Arm Lift.

Table S17. Average difference in muscle thickness between groups Sitting- VALSALVA.

		**Score difference**				**ANCOVA**	
		**Cases (n=26)**	**Controls (n=34)**	**p**	**Effect size**	**p**	**Effect size**
**Diaphragm Excursion (mm)**							
**Rest**	Median (IQR)	6.80 (4.85)	5.86 (3.62)	0.279a	0.140b	-	-
**During test**	Median (IQR)	8.10 (11.83)	5.42 (9.33)	0.387a	0.112b	-	-
**Pelvic Floor**							
**excursion(mm)**	Median (IQR)	2.83 (2.91)	2.24 (3.33)	0.536a	0.080b	-	-
**Rest**	Median (IQR)	-2.77 (13.63)	-0.72 (17.26)	0.748a	0.041b	-	-
**Activation**							
**Extenal Oblique (mm)**							
**Rest**	Median (IQR)	16.41 (16.26)	15.77 (14.99)	0.870a	0.021b	-	-
**Activation**	Median (IQR)	14.72 (12.77)	14.65 (15.12)	0.777a	0.037b	-	-
**Ratio**	Median (IQR)	0 (27.28)	0.01 (35.06)	0.858a	0.023b	-	-
**Internal Oblique**							
**(mm)**	Median (IQR)	21.84 (18.53)	14.99 (16.42)	0.177a	0.174b	-	-
**Rest**	Median (IQR)	25.03 (23.68)	18.15 (17.60)	0.465a	0.094b	-	-
**Activation**	Median (IQR)	11.42 (27.34)	7.80 (49.31)	0.911a	0.014b	-	-
**Ratio**							
**Transversus Abdominis (mm)**							
**Rest**	Mean (SD)	11.61 (5.22)	13.27 (6.48)	0.292c	0.279d	0.35	0.015f
**Activation**	Median (IQR)	17.00 (18.28)	16.55 (16.09)	0.602a	0.067b	4e	-
**Ratio**	Median (IQR)	41.69 (120.14)	28.12 (55.16)	0.175a	0.175b	-	-
						-	
**Lumbar Mutifidus (mm)**							
**Rest**	Mean (SD)	67.56 (30.44)	70.56 (30.05)	0.704c	0.100d	0.25	0.023f
**Activation**	Mean (SD)	70.83 (31.12)	75.23 (32.87)	0.601c	0.138d	9e	0.019f
**Ratio**	Median (IQR)	3.77 (7.04)	4.25 (8.27)	0.800a	0.033b	0.30	-
						1e	

-

aMann–Whitney U test, b Rosenthal's r, c Unpaired t-test, d Cohen's d, e ANCOVA, f Partial Eta-squared.

Table S18. Mean difference between groups in muscle thickness at Sitting– VPFC

		Score difference				ANCOVA	
		**Cases (n=26)**	**Controls (n=34)**	**P**	**Effect size**	**p**	**Effect size**
**Diaphragm Excursion (mm)**							
**Rest**	Mean (SD)	8.00 (4.71)	6.28 (4.02)	0.134a	0.398b	**0.018c**	**0.096d**
**Activation**	Mean (SD)	7.14 (9.71)	3.14 (6.04)	0.055a	0.514b	0.056c	0.064d
**Pelvic Floor excursion (mm)**							
**Rest**	Median (IQR)	3.11 (4.06)	2.11 (4.57)	0.101e	0.212f	-	-
**Activation**	Median (IQR)	12.67 (15.14)	8.26 (10.25)	0.152e	0.185f	-	-
**External Oblique (mm)**							
**Rest**	Median (IQR)	17.38 (14.32)	14.50 (19.64)	0.451e	0.097f	-	-
**Activation**	Median (IQR)	17.59 (10.98)	13.91 (16.11)	0.800e	0.033f	-	-
**Ratio**	Median (IQR)	-1.65 (19.96)	-2.41 (22.17)	0.698e	0.050f	-	-
**Internal Oblique (mm)**							
**Rest**	Median (IQR)	17.79 (23.44)	15.74 (20.10)	0.194e	0.168f	-	-
**Activation**	Median (IQR)	22.56 (23.71)	15.13 (20.11)	0.161e	0.181f	-	-
**Ratio**	Median (IQR)	14.68 (32.57)	12.70 (29.94)	0.698e	0.050f	-	-
**Transversus Abdominis (mm)**	Mean (SD)	12.35 (6.71)	12.78 (7.74)	0.821a	0.059b	0.424c	0.011d
**Rest**	Median (IQR)	17.20 (13.94)	11.44 (15.81)	0.276e	0.141f	-	-
**Activation**	Median (IQR)	23.37 (95.08)	23.98 (67.73)	0.988e	0.002f	-	-
**Ratio**							
**Lumbar Mutifidus (mm)**							
**Rest**	Mean (SD)	65.69 (24.05)	67.42 (29.32)	0.807a	0.064b	0.908c	0.001d
**Realización**	Mean (SD)	67.34 (25.25)	70.53 (30.30)	0.667a	0.113b	0.902c	0.001d
**Test**	Median (IQR)	1.56 (7.59)	1.62 (6.77)	0.416e	0.105f	-	-
**Ratio**							

a Unpaired t-test, b Cohen's d, c ANCOVA, d Partial Eta-squared, e Mann–Whitney U test, f Rosenthal's r. VPFC: Voluntary Pelvic Floor Contraction.
